# Hantaviruses in the Czech Republic

**DOI:** 10.3201/eid0906.020772

**Published:** 2003-06

**Authors:** Milan Pejčoch, Bohumír Kříž

**Affiliations:** * National Institute of Public Health, Prague, Czech Republic

**To the Editor:** Infections caused by hantaviruses have been known for a long time, but their causative agent was not detected until 1976 (*1*). These viruses of the genus Hantavirus, family Bunyaviridae, have >20 genotypes. Hantaviruses circulate in wild rodents within natural foci over Euroasia and North and South America. They cause asymptomatic persistent infections in these small mammals. Humans may acquire infection accidentally from inhalation of virus-contaminated aerosols of rodent excreta. Hantavirus genotypes may be nonpathogenic for humans or cause serious diseases with high death rates. In Eurasia, these pathogens involve primarily the kidney and cause hemorrhagic fever with renal syndrome; in North and South America, these pathogens involve primarily the lung and cause hantavirus cardiopulmonary syndrome.

First reports on the occurrence of hantaviruses in central Europe originated from former Czechoslovakia (*2*,*3*) and Germany (*4*) and date back to 1984 and 1985, respectively. The first cases of hantavirus disease in humans in the Czech Republic were reported in 1992 (*5*). This flulike disease accompanied by microhematuria was recorded in southern Moravia. Another severe imported case was described in a soldier on active military duty in the Balkans (*6*). The first isolation of nonpathogenic hantavirus Tula was reported in the Czech Republic (*7*). Currently, several hantavirus infections have been recorded in humans, manifesting mainly as interstitial nephritis. One fatal case was also reported in a patient who had never travelled outside the Czech Republic.

We conducted studies of hantavirus ecology in the Czech Republic and hantavirus seroprevalence in the Czech population. As in neighboring Slovakia (*8*), hantaviruses of three genotypes, i.e., Dobrava, Puumala, and Tula, were identified in the Czech Republic. Most serious infections are caused by the Dobrava genotype; Tula genotype remains nonpathogenic for humans, although a case of human infection without clinical signs has been described in the Czech Republic (*9*).

Blood serum samples from 710 randomly selected persons >20 years of age from the Czech Republic were screened for antibodies against Puumala and Hantaan antigens with commercial enzyme-linked immunosorbent assay (ELISA) sets manufactured by PROGEN (Biotechnik, Heidelberg, Germany). The Hantaan antigen was used because of its antigenic relatedness with Dobrava virus, which is not included in available commercial ELISA sets.

Five participants showed immunoglobulin (Ig) G reactivity to Hantaan virus (cross-reactive with Dobrava antigen), and two participants tested positive for both IgG and IgM antibodies. Two other persons showed IgM reactivity alone. These findings indicate that as many as seven (1.0%) study participants showed reactivity to Hantaan antigen. Eight persons showed IgG reactivity to Puumala antigen, none of them IgM positive. Altogether, 10 (1.4%) study participants were reactive to Puumala antigen. Three persons showed reactivity to both antigens tested.

A total of 1,494 small mammals of different Czech regions were screened with ELISA for hantavirus antigen in the lungs. The antigen was detected in the lungs of 101 animals; the highest positivity rate was in Common Voles (Microtus arvalis). The difference in positivity between male and female voles was not statistically significant. The positivity rate was markedly associated with rodent size. With the use of molecular genetic methods (polymerase chain reaction), genotype Tula was identified as the causative agent of infection in rodents. Genotype Puumala was identified in Bank Voles (Clethrionomys glareolus) in Moravia. Nucleotide sequences of Dobrava genotype were identified in southern Bohemian rodents (K. Křivanec, pers. comm.).

In the Czech Republic, Tula virus is the most frequent hantavirus circulating in Common Voles. This agent is not pathogenic for humans. The hantavirus seroprevalence rate in the adult population of the Czech Republic is close to 1%. Dobrava and Puumala viruses are causative agents of these infections in humans.
